# Effects of Pomegranate Juice on Androgen Levels, Inflammation and Lipid Profile in Polycystic Ovary Syndrome: A Systematic Review and Meta-Analysis

**DOI:** 10.3390/jcm14155458

**Published:** 2025-08-03

**Authors:** Vitória Silveira, Pamela Braz, Antonio Jose Grande, Tamy Colonetti, Maria Laura Rodrigues Uggioni, Gabriele da Silveira Prestes, Leonardo Roever, Valdemira Santina Dagostin, Maria Inês da Rosa

**Affiliations:** 1 School of Medicine, Universidade do Extremo Sul Catarinense (UNESC), Criciúma 88806-000, SC, Brazil; vitoriapsilveira010@gmail.com (V.S.); pamela.braz@unesc.net (P.B.); 2 School of Medicine, Universidade Estadual de Mato Grosso do Sul (UEMS), Campo Grande 79804-970, MS, Brazil; grandeto@gmail.com; 3 Translational Biomedicine Laboratory, Graduate Program in Health Sciences, Universidade do Extremo Sul Catarinense (UNESC), Criciúma 88806-000, SC, Brazil; tamycolonetti@unesc.net (T.C.); lala.uggioni@unesc.net (M.L.R.U.); gabrielesprestes@outlook.com (G.d.S.P.); vsd@unesc.net (V.S.D.); mir@unesc.net (M.I.d.R.); 4 Department of Clinical Research, Brazilian Evidence-Based Health Network, Uberlândia 38400-384, MG, Brazil

**Keywords:** pomegranate juice, polycystic ovary syndrome, hyperandrogenism, systematic review, meta-analysis

## Abstract

**Background/Objectives:** Polycystic ovary syndrome (PCOS) is a multifactorial endocrine disorder frequently associated with metabolic and inflammatory disturbances. Due to its antioxidant and anti-inflammatory properties, pomegranate juice has been proposed as a potential adjunctive therapy in managing PCOS. To evaluate the effects of pomegranate juice on hormonal, inflammatory, and lipid parameters and body mass index (BMI) in women with PCOS. **Methods:** A systematic review and meta-analysis of randomized controlled trials (RCTs) was conducted following PRISMA guidelines. Comprehensive searches were performed in electronic databases including Medline, Scopus, Web of Science, Cochrane CENTRAL, and Embase from inception to July 2025, using keywords and MeSH terms related to “polycystic ovary syndrome” and “pomegranate juice” without language restrictions. The primary outcomes were changes in serum testosterone, luteinizing hormone (LH), high-sensitivity C-reactive protein (hs-CRP), lipid profile parameters (HDL, LDL, triglycerides, and total cholesterol), and body mass index (BMI). **Results:** Four RCTs published between 2020 and 2023, encompassing 128 women with PCOS, were included. The meta-analysis revealed significant reductions in testosterone (MD: −0.05; 95% CI: −0.07 to −0.03; *p* < 0.0001; I^2^ = 0%, two studies, 85 participants) and hs-CRP (SMD: −0.85; 95% CI: −1.35 to −0.35; *p* = 0.0009; I^2^ = 20%, two studies, 85 participants), along with increases in HDL (MD: 6.21; 95% CI: 2.43 to 10.00; *p* = 0.001; I^2^ = 0%, two studies, 85 participants) and reductions in triglycerides (MD: −23.30; 95% CI: −45.19 to −1.42; *p* = 0.04; I^2^ = 0%, two studies, 85 participants). No significant changes were observed in LH, LDL, total cholesterol, or BMI. **Conclusions:** Pomegranate juice demonstrates promising effects as an adjunctive intervention in women with PCOS, improving androgen levels, inflammatory markers, and certain lipid parameters. Further long-term studies are needed to confirm these findings.

## 1. Introduction

Polycystic ovary syndrome (PCOS) represents one of the most common endocrine disease affecting women of reproductive age. It is primarily defined by the presence of hyperandrogenism, ovulatory irregularities, and polycystic ovarian morphology [1,2,3]. Beyond its gynecological implications, PCOS is frequently linked with a constellation of metabolic disturbances, including but not limited to insulin resistance, dyslipidemia, endothelial dysfunction, and persistent low-grade inflammation. These metabolic comorbidities substantially elevate the risk for long-term cardiovascular and metabolic diseases [4,5]. The syndrome’s etiological and clinical heterogeneity continues to complicate both its diagnostic criteria and therapeutic approaches, underscoring the need for sustained scientific inquiry [6,7].

Diagnostic classification, as per the Rotterdam criteria, mandates the presence of at least two out of three features—namely oligo- or anovulation, biochemical or clinical hyperandrogenism, and polycystic ovarian morphology as identified via ultrasonography—after the exclusion of alternative causes [8].

Pharmacological management frequently incorporates the use of combined oral contraceptives to normalize menstrual function and attenuate androgen excess [9]. Additionally, insulin-sensitizing agents such as metformin are widely employed to enhance insulin responsiveness, stabilize glycemic profiles, reduce circulating androgens, and improve lipid metabolism [10].

Alongside medical interventions, non-pharmacological strategies—particularly those centered on dietary modification and increased physical activity—have become integral components of PCOS care, aimed at addressing associated metabolic and cardiovascular risks [11]. Emerging evidence implicates gut microbiota dysbiosis and increased intestinal permeability as potential contributors to the chronic inflammatory state observed in PCOS [12].

In light of these findings, there has been growing interest in the therapeutic potential of dietary interventions enriched with functional foods containing high concentrations of antioxidants and bioactive constituents. Among such candidates, pomegranate (*Punica granatum* L.) has garnered particular attention due to its rich profile of polyphenols, ellagitannins, flavonoids, and antioxidative vitamins [13,14]. Its pharmacodynamic properties are believed to include anti-inflammatory and antioxidant effects alongside vasoprotective actions mediated via nitric oxide bioavailability and the inhibition of cyclooxygenase pathways [15].

Although various populations have demonstrated favorable outcomes with pomegranate supplementation, data specific to its impact on hormonal, metabolic, and inflammatory indices in women with PCOS remain limited and inconclusive [16,17,18,19]. Given the complex and multifactorial nature of PCOS, coupled with the shortcomings of current treatment modalities, this review endeavors to critically appraise and synthesize existing randomized controlled trials that investigate the effects of pomegranate juice on key clinical endpoints in this patient group.

To our knowledge, this is the first systematic review and meta-analysis to focus specifically on the effects of pomegranate juice—excluding extracts or encapsulated formulations—as a nutritional intervention for women with PCOS.

## 2. Methods

A systematic review was conducted in accordance with the PRISMA (Preferred Reporting Items for Systematic Reviews and Meta-Analyses) guidelines [20]. The protocol was prospectively registered with the PROSPERO database (International Prospective Register of Systematic Reviews), under the registration number CRD42024570356, thereby ensuring transparency and methodological rigor. The investigation was carried out within the Translational Biomedicine Laboratory, affiliated with the Graduate Program in Health Sciences at the Universidade do Extremo Sul Catarinense.

### 2.1. PICO Framework

This review was structured according to the PICO model, a cornerstone of evidence-based practice (EBP) that facilitates the formulation of focused clinical questions and the development of comprehensive search strategies [21,22,23,24,25,26]. The inclusion criteria were as follows:

- Population: Women diagnosed with polycystic ovary syndrome (PCOS)
- Intervention: Pomegranate juice
- Comparison: Placebo
- Outcomes: Hormonal parameters (testosterone, luteinizing hormone), inflammatory marker (high-sensitivity C-reactive protein, hs-CRP), lipid profile (low-density lipoprotein, high-density lipoprotein, triglycerides, and total cholesterol), and body mass index (BMI)

### 2.2. Inclusion and Exclusion Criteria

Eligible studies were randomized controlled trials investigating the effects of pomegranate juice in women with PCOS, using placebo as a control comparator. Studies were excluded if they involved participants with comorbid conditions known to confound androgen levels or otherwise failed to meet the specified inclusion criteria.

### 2.3. Search Strategy

A systematic search was conducted in the databases MEDLINE via PubMed, EMBASE via Elsevier, Cochrane Library, LILACS via BVS, and gray literature. The search strategy incorporated the terms “polycystic ovary syndrome” and “pomegranate”, “pomegranate juice”, “pomegranate extract”, along with relevant synonyms derived from the Medical Subject Headings (MeSH) database up to 24 July 2025. Boolean operators “OR” and “AND” were applied to broaden the search scope. No language restrictions were imposed. Additionally, reference lists of the retrieved articles were manually screened for potential supplementary studies.

The following search strategy was applied:

- MEDLINE (via PubMed): (“polycystic ovary syndrome” OR “PCOS” OR “Stein-Leventhal Syndrome”) AND (“pomegranate juice” OR “pomegranate extract” OR “pomegranate peel” OR “pomegranate seed” OR “Punica granatum” OR “pomegranate supplement” OR “pomegranate powder” OR “pomegranate formulation”).
- EMBASE (via Elsevier): (‘ovary polycystic disease’/exp OR ‘ovary polycystic disease’) AND (‘pomegranate’/exp OR ‘pomegranate’ OR ‘pomegranate extract’/exp OR ‘pomegranate extract’ OR ‘pomegranate juice’/exp OR ‘pomegranate juice’).
- LILACS (via BVS): síndrome dos ovários policísticos OR polycystic ovary syndrome OR síndrome de ovário poliquístico) AND pomegranate juice OR suco de romã OR romã OR jugo de granada OR granada OR pomegranate extract OR extrato de romã OR semente de romã.
- Cochrane Library: “polycystic ovary syndrome” AND “pomegranate” OR “pomegranate extract” OR “pomegranate juice”.
- Gray Literature: (Google scholar; ProQuest Dissertations and Theses; SIGLE): “polycystic ovary syndrome” AND “pomegranate” OR “pomegranate extract” OR “pomegranate juice”.

### 2.4. Study Selection

Identified records were imported into the Rayyan platform (https://www.rayyan.ai/ accessed on 21 March 2024) (for title and abstract screening. Two reviewers (V.P.S. and P.S.B.) independently assessed each entry for relevance to the eligibility criteria. Discrepancies were resolved through discussion or, when necessary, adjudicated by a third reviewer (M.I.R.).

### 2.5. Data Extraction

Data extraction was performed independently by two reviewers (V.P.S. and P.S.B.) using a standardized form that recorded key study attributes: author(s), year of publication, country of origin, study objectives, participant characteristics, methodological design, intervention specifics, and reported outcomes. Disagreements were addressed through consultation with a third reviewer (M.I.R.).

### 2.6. Risk of Bias and Quality of Evidence Assessment

The methodological quality of each included study was appraised using the Cochrane Risk of Bias 2 (RoB 2) tool [27], which evaluates five domains of potential bias: (1) bias arising from the randomization process; (2) bias due to deviations from intended interventions; (3) bias due to missing outcome data; (4) bias in measurement of the outcome; and (5) bias in selection of the reported result. Each domain was rated as “Low,” “High,” or “Some concerns” using structured signaling questions. Evaluation was conducted independently by two reviewers in accordance with the Cochrane Handbook.

The certainty of evidence for each outcome was assessed using the GRADE approach via GRADEpro (www.gradepro.org), with an initial classification of “High” for randomized trials. This rating was subsequently downgraded based on risk of bias, inconsistency, indirectness, imprecision, or suspected publication bias.

### 2.7. Statistical Analysis

Effect sizes for continuous outcomes were reported as mean differences (MDs) or standardized mean differences (SMDs), depending on measurement scales across studies. All estimates were presented with 95% confidence intervals (CIs).

Meta-analyses were conducted using Review Manager (RevMan) version 5.4. Heterogeneity was quantified using the I^2^ statistic and interpreted as follows: 0–30% (low), 30–60% (moderate), 60–90% (substantial), and >90% (considerable). A random-effects model (DerSimonian and Laird method) was employed [28].

Forest plots were generated to visualize effect sizes across studies. A *p*-value less than 0.05 was considered statistically significant for pooled estimates.

## 3. Results

**Figure 1:**
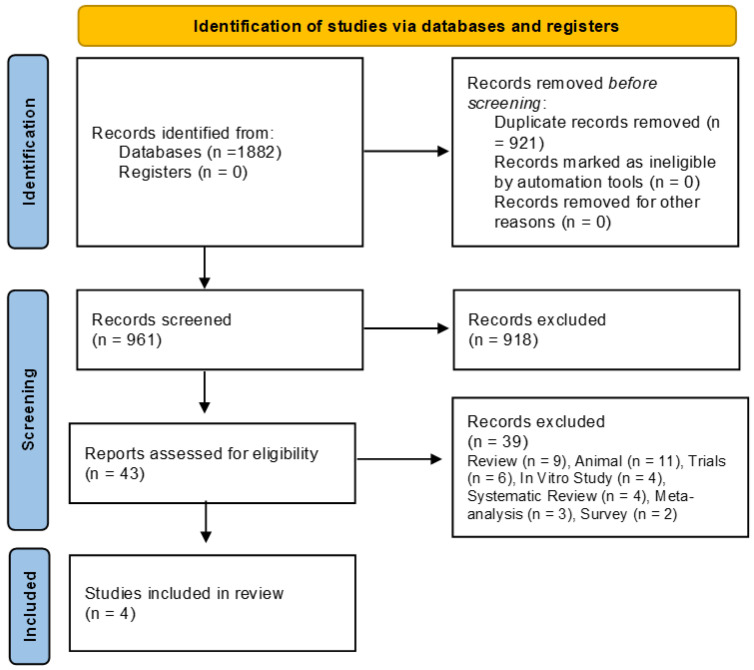
Selection of included studies.

Following the execution of the database search strategy, a total of 1882 studies were identified. Of these, 921 were excluded due to duplication, leaving 961 articles for title and abstract screening. Subsequently, 918 articles were excluded for not meeting the eligibility, leaving 43 for full-text reviews; finally, 39 articles were excluded (review (*n* = 9), animal (*n* = 11), clinical trial registration (*n* = 6), in vitro study (*n* = 4), systematic review (*n* = 4), meta-analysis (*n* = 3), and survey (*n* = 2), resulting in four eligible studies. Therefore, four studies were included in this systematic review and meta-analysis [16,17,18,19]. The selection process is illustrated in the flowchart (Figure 1).

### 3.1. Characteristics of the Included Studies

The four studies analyzed were conducted in Iran between 2018 and 2023, including a total of 128 participants. All participants were women aged between 15 and 48 years, previously diagnosed with PCOS according to the Rotterdam criteria. Participants did not present other medical conditions associated with hyperandrogenism and were all classified as overweight, with a BMI greater than 25 kg/m^2^. Exclusion criteria included pregnancy, lactation, menopause, smoking, and the use of medications such as antidiabetics, contraceptives, corticosteroids, anti-inflammatories, or supplements. Additionally, individuals with chronic diseases (e.g., diabetes, hypertension, cardiovascular disease, thyroid disorders, depression, or Cushing’s syndrome) or allergies to pomegranate were excluded. The general characteristics of the included studies are detailed in Table 1.

The studies by Abedini et al. [16,17] were designed as randomized controlled trials (RCTs). Participants were assigned to either the intervention group, which received pomegranate juice, or the control group. Allocation was stratified based on age and metformin use, and randomization within each stratum was conducted by a researcher blinded to the study objectives to ensure impartiality. The intervention group received 45 mL of pomegranate juice diluted in 180 mL of water daily for eight weeks. Juice was distributed in three batches (baseline, week 3, and week 6), and compliance was monitored through the return of empty bottles. The control group was instructed to avoid pomegranate consumption throughout the study period.

The studies conducted by Esmaeilinezhad et al. [18,19] were triple-blinded randomized controlled trials. Participants were randomly allocated into four equal groups, each receiving a different beverage over eight weeks: Group 1 (synbiotic pomegranate juice with inulin and lactobacilli), Group 2 (pure pomegranate juice), Group 3 (synbiotic beverage containing water, inulin, lactobacilli, and pomegranate flavor), and Group 4 (placebo beverage composed of water with pomegranate flavor). Randomization was performed using specialized software without researcher involvement. To maintain blinding, all beverages had similar appearance, color, and taste, and were coded. Blood samples were collected before and after the intervention, and dietary questionnaires were applied to monitor compliance. Table 2 presents the meta-analysis outcomes.

### 3.2. Meta-Analysis Outcomes

#### 3.2.1. Testosterone

**Figure 2:**
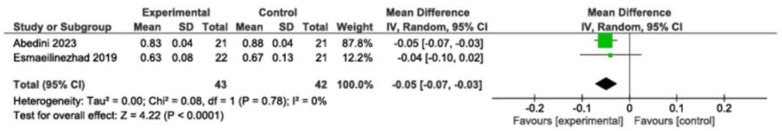
Meta-analysis of testosterone levels [17,19]. Legend: Green squares represent individual study estimates, with horizontal lines indicating their 95% confidence intervals. The black diamond represents the overall pooled effect estimate and its 95% confidence interval.

Decreased significantly (MD: −0.05; 95% CI: −0.07 to −0.03; *p* < 0.0001; I^2^ = 0%, two studies, 85 participants). High-quality evidence (Figure 2).

#### 3.2.2. hs-CRP

**Figure 3:**
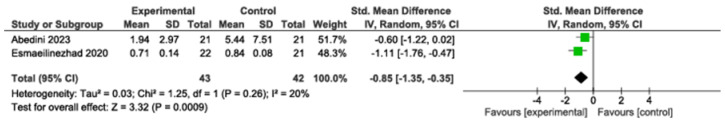
Meta-analysis of hs-CRP levels [17,18]. Legend: Green squares represent individual study estimates, with horizontal lines indicating their 95% confidence intervals. The black diamond represents the overall pooled effect estimate and its 95% confidence interval.

Decreased significantly (SMD: −0.85; 95% CI: −1.35 to −0.35; *p* = 0.0009; I^2^ = 20%, two studies, 85 participants). High-quality evidence (Figure 3).

#### 3.2.3. HDL

**Figure 4:**
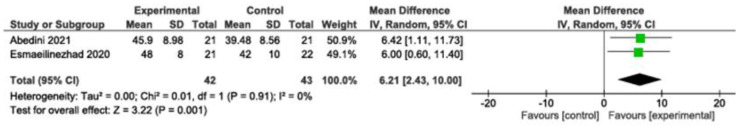
Meta-analysis of HDL levels [16,18]. Legend: Green squares represent individual study estimates, with horizontal lines indicating their 95% confidence intervals. The black diamond represents the overall pooled effect estimate and its 95% confidence interval.

Increased significantly (MD: 6.21; 95% CI: 2.43 to 10.00; *p* = 0.001; I^2^ = 0%, two studies, 85 participants). High-quality evidence (Figure 4).

#### 3.2.4. Triglycerides

**Figure 5:**
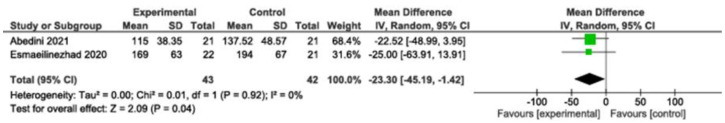
Meta-analysis of triglyceride levels [16,18]. Legend: Green squares represent individual study estimates, with horizontal lines indicating their 95% confidence intervals. The black diamond represents the overall pooled effect estimate and its 95% confidence interval.

Decreased significantly (MD: −23.30; 95% CI: −45.19 to −1.42; *p* = 0.04; I^2^ = 0%, two studies, 85 participants). Moderate-quality evidence (Figure 5).

#### 3.2.5. LH, LDL, Total Cholesterol, BMI

Not significant. The complete data from the analysis are available in Figures S1–S3 (Supplementary Materials).

### 3.3. Risk of Bias

The Cochrane RoB 2 tool was used to assess risk of bias. Esmaeilinezhad et al. [18,19] and Abedini et al. [16,17] presented low risk of bias in randomization (Domain 1). In Domain 2 (deviations from intended interventions), Esmaeilinezhad et al. [18,19] maintained blinding using coded, identical beverages, resulting in low risk of bias. Conversely, Abedini et al. [16,17] lacked placebo controls, which may have led participants to discern their group allocation; however, strict monitoring protocols maintained a low risk of bias. Intention-to-treat analysis was applied in Esmaeilinezhad et al. [18,19] but not in Abedini et al. [16,17]. Nonetheless, loss to follow-up was related to participants initiating other medical therapies, not biasing the outcome (Domain 3).

**Figure 6:**
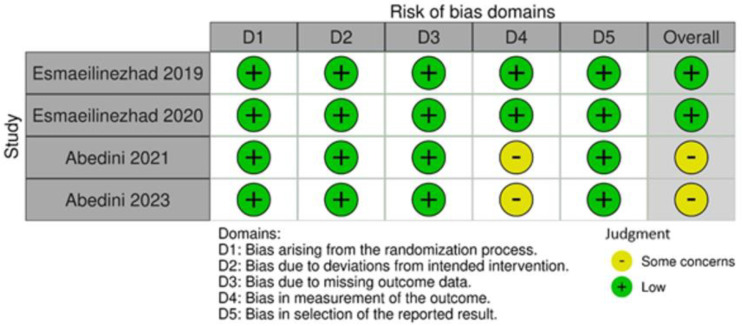
Risk of bias of the included studies [16,17,18,19].

For Domain 4 (blinding of outcome assessors), Esmaeilinezhad et al. [18,19] maintained low risk, while Abedini et al. [16,17] were rated as “some concerns” due to lack of clarity regarding outcome assessor blinding. In Domain 5 (selection of the reported result), all studies adhered to predefined protocols, resulting in low risk of bias. Thus, overall risk was classified as “some concerns” for Abedini et al. [16,17] and as “low” for Esmaeilinezhad et al. [18,19]. This is illustrated in Figure 6.

### 3.4. Quality of Evidence Assessment

The GRADE assessment is summarized in Table 3. The quality of evidence was rated as high for testosterone, LH, hs-CRP, lipid profile, and BMI outcomes. However, triglycerides and LDL were downgraded to moderate quality due to imprecision related to wide confidence intervals and null effect overlaps. Other outcomes maintained high certainty of evidence.

## 4. Discussion

This systematic review evaluated the effects of pomegranate juice in women with polycystic ovary syndrome (PCOS), focusing on hormonal, inflammatory, and lipid markers as well as body mass index (BMI). The results demonstrated a significant reduction in testosterone levels, suggesting a potential antiandrogenic effect. Improvements were also observed in inflammatory markers and lipid profiles, particularly in reductions in triglycerides and high-sensitivity C-reactive protein (hs-CRP), alongside an increase in high-density lipoprotein (HDL). However, no significant changes were observed in low-density lipoprotein (LDL), total cholesterol, luteinizing hormone (LH) levels, or BMI.

Pomegranate extract appears to exert an indirect antiandrogenic effect due to its phytoestrogen content, whose molecular structures resemble that of endogenous estrogen. These compounds may compete for estrogen receptors, leading the body to interpret estrogen levels as adequate. This, in turn, activates a negative feedback mechanism in the hypothalamic–pituitary–ovarian axis, resulting in reduced LH secretion and subsequently lowered testosterone production [29,30]. Since this mechanism modulates secretion rather than directly reducing circulating LH levels, the effect on LH may be insufficiently strong to reach statistical significance—consistent with the findings of this review. In contrast, the significant reduction in testosterone supports the hypothesis that pomegranate juice can effectively reduce androgen levels compared with placebo. This aligns with findings from animal models, which observed similar effects in animal models of benign prostatic hyperplasia [31]. Furthermore, pomegranate derivatives might inhibit estrogen-sensitive breast cancer cell proliferation in vitro, highlighting broader implications for hormone-related conditions [32].

Regarding hs-CRP, pomegranate juice significantly reduced levels compared with placebo. This is likely attributable to its phytochemical content, particularly ellagitannins, which directly inhibit nuclear factor kappa B (NF-κB). NF-κB regulates pro-inflammatory cytokines such as interleukin-6 (IL-6) and tumor necrosis factor-alpha (TNF-α), which in turn stimulate hepatic hs-CRP production [33,34]. These results are consistent with findings from a systematic review of 16 randomized controlled trials (RCTs)—seven involving pomegranate supplementation—reported significant hs-CRP reductions (*p* = 0.000), especially in female participants and interventions under eight weeks [35]. Reduced hs-CRP is a clinically meaningful indicator of improved vascular health, given its association with endothelial dysfunction and subsequent cardiovascular risk [36].

In terms of lipid metabolism, pomegranate juice appears to act primarily through the activation of peroxisome proliferator-activated receptor alpha (PPAR-α) [37,38]. This may explain the significant reductions in triglycerides and increases in HDL observed in this review, despite no significant changes in LDL or total cholesterol. The lack of effect on LDL may stem from pomegranate’s limited influence on hepatic LDL receptor expression or methodological issues in the included studies, such as brief intervention durations, dosing variability, and juice formulation differences. This interpretation is consistent with a systematic review and meta-analysis of randomized controlled trials, which found no significant reductions in oxidized LDL across four RCTs, although a downward trend was noted [39]. Moreover, a systematic review and meta-analysis of randomized controlled clinical trials highlighted that pomegranate’s bioavailability may be constrained by multiple factors, limiting its efficacy on certain lipid parameters [40].

It is also important to consider that total cholesterol reflects the combined values of its individual fractions, as calculated by the Friedewald formula [41]. Therefore, despite increased HDL and decreased triglycerides, the stability of LDL levels likely contributed to the lack of significant change in total cholesterol. While elevated HDL is beneficial—especially given the heightened cardiovascular risk in PCOS patients—it does not confer complete protection in the absence of LDL control [42]. Furthermore, reductions in triglycerides are closely linked to improved insulin sensitivity in PCOS. Hyperinsulinemia exacerbates the condition by stimulating LH receptors, inhibiting aromatase activity, and lowering sex hormone-binding globulin (SHBG) levels, thereby perpetuating the syndrome’s pathophysiology [43]. 

Regarding BMI, no statistically significant changes were detected between the intervention and control groups. This may be due in part to the short eight-week intervention period, which might be inadequate for detecting meaningful weight changes. Additionally, although participants were advised to maintain their usual dietary and lifestyle habits, it remains unclear whether these habits were conducive to weight loss. The lack of structured physical activity interventions—which independently contribute to BMI reduction—may also have influenced these results [44]. This finding is consistent with findings from a systematic review across various populations—including healthy, overweight/obese individuals, and patients with chronic conditions—found no significant effects of pomegranate on body weight or composition, possibly because weight was not the primary outcome in most studies [45]. Nonetheless, pomegranate is rich in polyphenols, flavonoids, and dietary fibers—especially concentrated in the peel—that may aid in reducing waist circumference, waist-to-hip and waist-to-height ratios, and body fat percentage, as demonstrated in a double-blind, randomized, placebo-controlled trial [46].

All four trials were carried out in Iran, a clustering that probably reflects a confluence of cultural, agronomic, and research-policy factors. Pomegranate (*Punica granatum* L.) is native to, and still extensively cultivated in, Iran and adjoining regions, making fresh fruit and standardized juice concentrates inexpensive and readily available for clinical research [47]. In the Persian system of traditional medicine, pomegranate has long been regarded as a women’s health remedy, which increases public acceptance and participant adherence in studies targeting conditions such as polycystic ovary syndrome (PCOS). Iranian medical universities actively promote investigator-initiated trials of low-cost complementary interventions, and several national funding calls specifically encourage natural-product research; this supportive infrastructure may explain why multiple, methodologically similar RCTs on pomegranate juice and PCOS have emerged from the same country [48]. While this geographic homogeneity enhances the internal consistency of our pooled estimates, it also limits external validity: replication in other cultural and dietary contexts—particularly in non-Middle Eastern populations with different pomegranate intake patterns—is needed before the findings can be generalized worldwide.

In addition to the indirect antiandrogenic effects attributed to the polyphenols and phytoestrogens present in pomegranate juice, it is important to consider the potential direct action of antioxidant compounds. Polyphenol-rich fractions from pomegranate seed oil and fermented juice significantly inhibited copper-induced LDL oxidation, highlighting the antioxidant capacity of these components and their potential role in reducing systemic oxidative stress—a factor potentially linked to androgen excess [49]. Furthermore, a network meta-analysis found that antioxidant agents such as vitamin E, coenzyme Q10, inositols, and vitamin D were effective in improving both endocrine and metabolic profiles in women with PCOS, including significant reductions in testosterone levels [50]. These findings support the hypothesis that the observed androgen-lowering effects of pomegranate juice may, at least in part, be related to its potent antioxidant properties, comparable to those of isolated micronutrients.

An additional source of heterogeneity among the included studies may stem from the seasonality and cultivar-specific variation in pomegranate composition. Iran, where all four trials were conducted, is known for diverse local cultivars that differ in polyphenol concentration, antioxidant capacity, and ellagitannin content. Moreover, harvest time and seasonal conditions (e.g., temperature, rainfall) can affect the maturity and biochemical profile of the fruit. Since none of the included studies provided detailed information about the cultivar or harvest period, these factors may have contributed to variations in biochemical potency.

To enhance methodological robustness, we reanalyzed all outcomes using random-effects models, regardless of the observed heterogeneity (I^2^ value), to account for potential between-study variability. This approach aligns with the clinical diversity present across studies and provides more conservative and generalizable estimates. The updated results are presented in the revised figures and tables, with no meaningful changes in the direction or significance of the primary outcomes.

The hypothesized antiandrogenic effect of pomegranate may be partly mediated by its phytoestrogen content, which includes compounds structurally similar to estradiol that are capable of competing for estrogen receptors. This interaction could trigger a negative feedback mechanism in the hypothalamic–pituitary–ovarian axis, reducing LH secretion and consequently lowering androgen production. While this mechanism has been demonstrated in vitro and in animal models [31,32], human evidence remains limited. Some clinical studies suggest that dietary phytoestrogens, such as soy isoflavones, may modulate androgen levels in women with PCOS [51,52]. However, the specific estrogenic activity of pomegranate phytoestrogens in humans requires further investigation.

Although the reduction in total testosterone was statistically significant (MD: −0.05 ng/mL; 95% CI: −0.07 to −0.03), the absolute change is modest and may not independently resolve clinical hyperandrogenism. However, even small reductions in androgen levels—when combined with other improvements such as reduced inflammation and enhanced lipid profile—may contribute to a clinically meaningful improvement in symptoms and cardiometabolic risk in women with PCOS. The observed 6.2 mg/dL increase in HDL and 23.3 mg/dL reduction in triglycerides, for example, are considered cardioprotective changes, particularly relevant in overweight women with PCOS who are at elevated cardiovascular risk.

### 4.1. Limitations

This meta-analysis presents several important limitations that warrant careful consideration when interpreting the findings. The analysis is constrained by the limited number of included randomized controlled trials and the relatively small total sample size across studies, which may reduce the statistical power to detect smaller but clinically meaningful effects. The short intervention durations employed in the included studies may not adequately capture the long-term physiological effects of pomegranate juice supplementation, particularly for parameters that require extended periods to demonstrate meaningful changes.

A significant methodological concern relates to the considerable variability in pomegranate juice composition across studies stemming from differences in fruit varieties, processing methods, storage conditions, and standardization protocols. This heterogeneity in bioactive compound concentrations makes it challenging to establish definitive dose–response relationships and may contribute to inconsistent findings. The absence of studies examining alternative consumption methods, such as incorporating pomegranate peel or other fruit components, represents a missed opportunity to explore potentially more effective delivery approaches.

Practical implementation challenges also emerge from the seasonal availability of fresh pomegranate, which is typically restricted to summer and spring months in most regions. This limitation poses significant obstacles for continuous supplementation protocols and may necessitate reliance on processed products or preservation methods that could alter the bioactive profile. Furthermore, the narrow participant demographics and limited geographic representation may restrict the generalizability of findings to broader populations with different baseline characteristics, dietary patterns, and genetic backgrounds.

### 4.2. Future Directions

Future research should prioritize larger-scale randomized controlled trials with extended follow-up periods to better establish both efficacy and safety profiles. Standardization of pomegranate preparations, including consistent bioactive compound concentrations and quality control measures, is essential for reproducible results. Investigation of optimal dosing strategies, treatment duration, and alternative delivery methods could enhance therapeutic potential while addressing practical implementation barriers. Additionally, research into preservation techniques or synthetic alternatives may help overcome seasonal availability constraints and support year-round supplementation strategies.

## 5. Conclusions

The findings of this systematic review suggest that pomegranate juice supplementation may offer beneficial effects on hormonal, inflammatory, and lipid profiles in overweight women with PCOS. Notably, it was associated with significant reductions in testosterone, hs-CRP, and triglycerides, as well as an increase in HDL levels. While no significant changes were observed in BMI, LDL, or total cholesterol, the evidence supports the potential of pomegranate juice as a complementary approach in the management of PCOS, particularly for improving androgen excess and cardiometabolic risk factors.

## Figures and Tables

**Table 1:** Characteristics of the included studies.

Author	Year	Country	Outline	Total No.	Group 1: Pomegranate Juice + Synbiotic + age	Group 2: Pomegranate Juice + age	Group 3:Synbiotic Age	Group 4: Control + Age	Pomegranate Juice Shot + Synbiotic	Pomegranate Juice Shot	Synbiotic Dose	Control Group Dose	Type of Placebo	Duration	General Objective
Esmaeilinezhad et al. [19]	2019	Iran	Randomized triple-blind controlled trial	86	22 30.04 ± 6.39	22 29.30 ± 7.46	21 29.52 ± 5.82	21 30.60 ± 7.43	2 L (pomegranate juice + 20 g insulin + 2 × 10^8^ CFU/g lactobacillus) per week	2 L (pure juice) per week	2 L (1 L water + 20 g insulin + 2 × 10^8^ lactobacillus) per week	2 L (1 L water + pomegranate flavouring) per week	Drink with water + pomegranate aroma per week	8 weeks	Effect of synbiotic pomegranate juice on glycemic indexes, sex hormone profile and anthropometric measurements in patients with PCOS
Esmaeilinezhad et al. [18]	2020	Iran	Randomized triple-blind controlled trial	86	22 30.04 ± 6.39	22 29.30 ± 7.46	21 29.52 ± 5.82	21 30.60 ± 7.43	300 mL/day (pomegranate juice + insulin 20 g/L +108 Lactobacillus)	300 mL/day of pomegranate juice	300 mL/day (water + inulin + Lactobacillus rhamnosus GG, Bacillus coagulans, and Bacillus indicus + pomegranate flavor and red dye)	300 mL/day (water containing pomegranate flavor and red dye)	Water containing pomegranate flavor and red dye	8 weeks	Effects of daily consumption of pomegranate juice, synbiotic drink, synbiotic pomegranate juice, and placebo on lipid profile, oxidative stress, inflammation, and blood pressure in women with PCOS
Abedini et al. [16]	2021	Iran	Randomized controlled trial	42	Not rated	21 24.76 (1.13)	Not rated	21 25.57 (1.09)	Not rated	45 mL/day pomegranate juice + 180 mL water	Not rated	Have not received a placebo or control treatment	Not rated	8 weeks	Effects of pomegranate juice concentrate (CPJ) intake on risk factors for cardiovascular disease (CVD) in women with PCOS
Abedini et al. [17]	2023	Iran	Randomized controlled trial	42	Not rated	21 24.76 (1.13)	Not rated	21 25.57 (1.09)	Not rated	45 mL/day of PJ concentrate + 180 mL of water	Not rated	Have not received a placebo or control treatment	Not rated	8 weeks	Effects of intake of pomegranate juice concentrate (PJ) on improving sex hormone levels, inflammation, and oxidative stress responses in PCOS patients

**Table 2:** Meta-analysis outcomes.

Parameter	Effect	Mean Difference (MD)/Standardized Mean Difference (SMD)	95% Confidence Interval	*p*-Value	I^2^	Studies	Participants	Evidence Quality
Testosterone	↓ Decreased	MD: −0.05	−0.07 to −0.03	<0.0001	0%	2	85	High
hs-CRP	↓ Decreased	SMD: −0.85	−1.35 to −0.35	0.0009	20%	2	85	High
HDL	↑ Increased	MD: 6.21	2.43 to 10.00	0.001	0%	2	85	High
Triglycerides	↓ Decreased	MD: −23.30	−45.19 to −1.42	0.04	0%	2	85	Moderate
LH	No change	-	-	NS	-	-	-	-
LDL	No change	-	-	NS	-	-	-	-
Total cholesterol	No change	-	-	NS	-	-	-	-
BMI	No change	-	-	NS	-	-	-	-

Legend: ↑ Indicates an increase; ↓ Indicates a decrease.

**Table 3:** GRADEpro evaluation of the evidence produced.

Certainty Assessment							No. of Patients		Effect		Degree of General Certainty of Evidence
No. of Studies	Study Design	Risk of Bias	Inconsistency	Indirect Evidence	Inaccuracy	Other Considerations	Pomegranate Juice for PCOS	Placebo	Relative (95% CI)	Absolute (95% CI)	
BMI											
2	Randomized controlled trials	Non-Grave	Non-Grave	Non-Grave	Non-Grave	None	43	42	27.97	MD 0.54 lower (1.92 lowest to 0.84 highest)	⨁⨁⨁⨁ High
Testosterone											
2	Randomized controlled trials	Non-Grave	Non-Grave	Non-Grave	Non-Grave	None	43	42	0.73	MD 0.05 Minor (0.07 Minor to 0.03 Minor)	⨁⨁⨁⨁ High
LH											
2	Randomized controlled trials	Non-Grave	Non-Grave	Non-Grave	Non-Grave	None	43	42	9.66-	MD 0.86 higher (0.37 lower to 2.09 higher)	⨁⨁⨁⨁ High
hs-CRP											
2	Randomized controlled trials	Non-Grave	Non-Grave	Non-Grave	Non-Grave	None	43	42		SMD 0.85 Lower (1.35 Lowest to 0.35 Lower)	⨁⨁⨁⨁ High
TG											
2	Randomized controlled trials	Non-Grave	Non-Grave	Non-Grave	Serious	None	43	42	142-	MD 23.3 minor (45.19 minor to 1.42 minor)	⨁⨁◯ Moderate
CT											
2	Randomized controlled trials	Non-Grave	Non-Grave	Non-Grave	Non-Grave	None	43	42	177.47-	MD 2.27 lower (13.19 lowest to 8.64 higher)	⨁⨁⨁⨁ High
LDL											
2	Randomized controlled trials	Non-Grave	Non-Grave	Non-Grave	Serious	None	43	42	101.74-	MD 0.14 lower (12.15 lower to 11.87 higher)	⨁⨁◯ Moderate
HDL											
2	Randomized controlled trials	Non-Grave	Non-Grave	Non-Grave	Non-Grave	None	42	43	46.95-	MD 6.21 highest (2.43 highest to 10 highest)	⨁⨁⨁⨁ High

## Data Availability

Not applicable.

## Supplementary Materials

The following supporting information can be downloaded at: https://www.mdpi.com/article/10.3390/jcm14155458/s1, Figure S1—Meta-analysis of LH levels. Figure S2—Meta-analysis of total cholesterol levels. Figure S3—Meta-analysis of LDL levels. Figure S4—Meta-analysis of body mass index. Table S1—PRISMA 2020 checklist.

## Abbreviations

BMI	Body mass index
CI	Confidence interval
EBP	Evidence-based practice
GRADE	Grading of Recommendations Assessment, Development and Evaluation
GRADEpro	GRADE profiler
HDL	High-density lipoprotein
hs-CRP	High-sensitivity C-reactive protein
IL-6	Interleukin-6
LDL	Low-density lipoprotein
LH	Luteinizing hormone
MD	Mean difference
MeSH	Medical Subject Headings
NF-κB	Nuclear factor kappa b
PCOS	Polycystic ovary syndrome
PICO	Population, Intervention, Comparison, Outcomes
PPAR-α	Peroxisome proliferator-activated receptor alpha
PRISMA	Preferred Reporting Items for Systematic Reviews and Meta-Analyses
PROSPERO	International Prospective Register of Systematic Reviews
RCT	Randomized controlled trial
RCTs	Randomized controlled trials
RevMan	Review Manager
RoB 2	Risk of Bias 2 (Cochrane tool)
SHBG	Sex hormone-binding globulin
SMD	Standardized mean difference
TNF-α	Tumor necrosis factor-alpha
